# Research on depression in Parkinson disease: A bibliometric and visual analysis of studies published during 2012–2021

**DOI:** 10.1097/MD.0000000000029931

**Published:** 2022-08-05

**Authors:** Yan Liu, Linlin Ding, Yunyan Xianyu, Shuke Nie, Jiying Yang

**Affiliations:** a Department of Nursing, Renmin Hospital of Wuhan University, Wuhan, China; b School of Nursing, Hubei University of Chinese Medicine, Wuhan, China; c Department of Neurology, Renmin Hospital of Wuhan University, Wuhan, China.

**Keywords:** bibliometric, depression, disease hotspots, Parkinson disease

## Abstract

**Background:**

The diagnosis and treatment rate of Parkinson disease (PD) with depression has a low diagnostic rate, and there is no consensus on the choice of treatment mode. This study evaluates the global research trends of scientific outputs related to depression in PD from multiple perspectives, using a bibliometric analysis and visualization tool to scientifically analyze the knowledge from the literature.

**Methods:**

Literature related to depression in PD published from 2012 to 2021 was included and selected from the Web of Science Core Collection database in October 2021. CiteSpace software was used to visualize and analyze co-occurrence analyses for countries, institutions, authors, and keywords.

**Results:**

A total of 4533 articles from the Web of Science database were included. The United States made the largest contribution with the majority of publications (1215; 29.40%). Toronto University was the most productive institution. PD, depression, quality of life, dementia, nonmotor symptom, prevalence, anxiety, Alzheimer disease, symptom, and disorder would be significantly correlated with depression in PD. The current hot spots in this field focus on the following: risk factors for depression in PD, assessment scale of depression in PD, and rehabilitation of depression in PD.

**Conclusions:**

This analysis not only reveals the current research trends and hotspots but also provides some instructive suggestions on the development of depression in PD.

## 1. Introduction

Parkinson disease (PD) is the second most common neurodegenerative disease after Alzheimer’s disease.^[1]^ Patients with PD present with both motor and nonmotor symptoms. In the motor system, PD is associated with static tremor, bradykinesia, muscle stiffness, and postural instability. In the nonmotor system, the main manifestations are cognitive changes, behavioral and neurological changes, pain and fatigue, autonomic nervous dysfunction, psychosis and hallucinations, sleep disorders, depression, and anxiety.^[2]^ Among them, depression is the most prominent and can appear in various stages of PD.^[3]^ Owing to inconsistencies in sampling procedures, assessment techniques, and definitions of depression, the estimated prevalence of depressive symptoms in Parkinson varies widely, ranging from 2.7% to 90%.^[4]^ Depression is associated with more severe disease and a lower quality of life in PD patients with.^[5]^ Tumor disease, no current partner, severe motor dysfunction, poor sleep quality, and anxiety are risk factors for PD with depression.^[6]^

Depression in PD patients can affect their function and quality of life. In a follow-up study of 353 patients over 7 years, those diagnosed with depression after PD were more likely to develop dementia than those diagnosed with depression before PD diagnosis (hazard ration = 2.01 [95% confidence interval 1.14–3.53]).^[7]^At the same time, people with patients present a greater burden for caregivers.^[8]^ Depression can increase fatigue, reduce motivation, further reduce independence and the ability to perform daily activities, and caregivers may worry about suicide. Although depression is common in patients with PD, it is often unrecognized and untreated. One study found that 27.6% of newly diagnosed PD patients had depression, but 40% were not evaluated or treated for depression.^[9]^

The prevalence of PD depression and its risk factors is high. However, the diagnosis and treatment rate of PD with depression are not high at present, and there is no consensus on the choice of treatment mode. Therefore, it is necessary to provide a comprehensive overview of research on PD over the past decade. Prevention of depression in PD can not only slow down the progression of PD but also reduce the burden on caregivers. Therefore, this study used the CiteSpace software system to comprehensively analyze the literature on depression in PD in the Web of Science Core Collection. To understand the research status and development trends of PD depression. Control of current hot research direction. It provides theoretical guidance for further studies on the factors influencing PD depression and prevention of PD depression.

## 2. Method

### 2.1. Data selected

The input data of this study were obtained using a combination of research results from multiple topic search queries in the Web of Science Core Collection. This study employed Medical Subject Headings and entry terms, either singularly or in combination.

First, we ensured that the data used were collected from 2012 to October 2021. The second places stress on Parkinson and depression. One of the topic terms included “Idiopathic Parkinson disease”, “Idiopathic Parkinson’s disease”, “LewyBody Parkinson’s disease”, “Parkinson’s disease, Idiopathic”, “Parkinson’s disease”, “Idiopathic Parkinson disease”, “Primary Parkinsonism”, “Paralysis Agitans”. Another topic term consists of “Depression*”, “Depressive Symptom*”, “Symptom*, Depressive”, “Depression*, Emotional”, and “Emotional Depression*”. This review is limited to the records of types of articles or reviews in English in the Web of Science Core Collection, containing 4533 records.

All bibliographic information was downloaded and saved as plain-text files for subsequent data processing and analysis. Subsequently, the data were imported into CiteSpace, and duplicate data were deleted to prepare for the next visualization step.

### 2.2. Data analysis method

CiteSpace is an information visualization tool extensively applied in the field of knowledge graphs.^[9]^ Visualization tools were adopted to display and analyze the knowledge context of a certain domain, and the development process and structural relationships in this domain were suggested. Therefore, this review adopted the CiteSpace5.8.R3 (64-bit) to achieve visualization to gain insights into this field of depression in PD and discover the research frontier and knowledge base of the field using considerable data.

Notably, when the clustering function was started, modularity Q and mean silhouette scores critically impacted the visualization, representing an overall structural characteristic of the network. Overall, Q > 0.3 displayed an overall significant structure. If S is > 0.5 or higher, the cluster is usually considered to be reasonable.^[10]^

Our research without directly relates to individual patients, and therefore, the issue of ethical review does not exist. Our research is a bibliographic analysis, mainly involving literature review and analysis.

## 3. Result

### 3.1. Analysis results and visualization

#### 3.1.1. Basic statistical analysis

**Figure 1.**
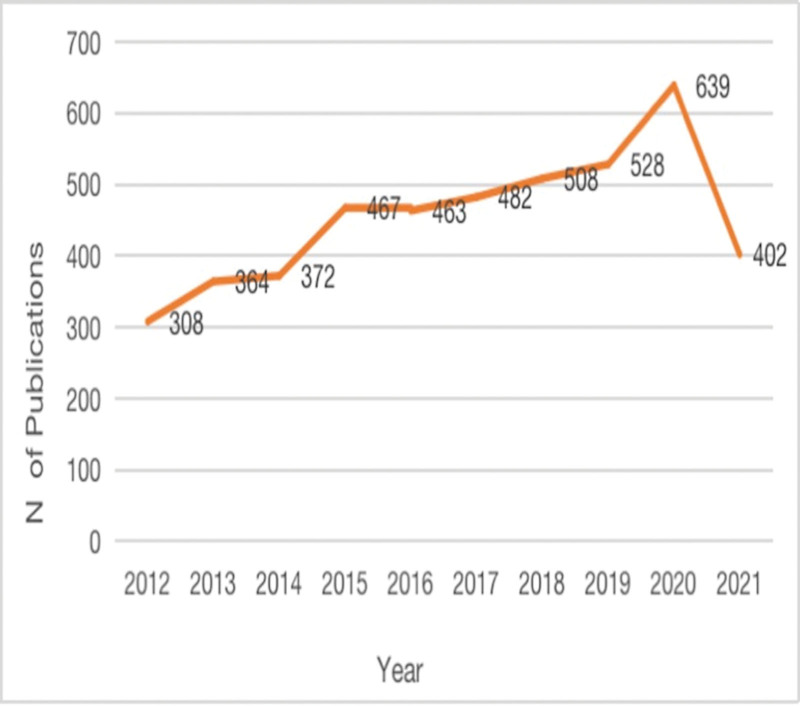
Studies regarding depression in Parkinson disease (2012–2021).

The number of studies published on falls in older adults has increased from 308 in 2012 to 402 in 2021 (Fig. 1). Depression in PD is attracting increasing attention from researchers. PD is one of the most common neurodegenerative diseases in the elderly population. It can be seen that attention in this field is gradually increasing, which may be related to the aging of the world population, the improvement of economic level, and the development of science and technology. Less research on depression in PD has been published in 2021 than in 2020. It is presumed that this decline may be due to coronavirus disease 2019.

#### 3.1.2. Co-country analysis

We ran CiteSpace, generating a network as usual: 2012–2021, slice length: 1 year; node selected the node type: country, top N = 50, choice pathfinder, pruning sliced networks, and pruning the merged network. The other parameters were set to default settings. In addition, co-country knowledge mapping was generated, in which N = 93 and E = 110 (density = 0.0257).

The analysis of the distribution of countries, institutions, and authors is to understand the current international scientific research center of depression in PD. The top10 countries, institutions, and authors in terms of publication outputs during 2012–2021 are listed in Table 1.

**Table 1:** The top 10 countries, institutions and authors in terms of publications included in WoS database during 2012–2021.

	**Publications, n (%**)	**Institution**	**Publications, n (%**)	**Author**	**Publications, n (%**)
United States	1215 (29.40)	University of Toronto	103 (14.74)	Daniel Weintraub	43 (15.20)
China	555 (13.43)	King College London	101 (14.45)	Pablo Martinezmartin	42 (14.84)
England	426 (10.31)	University of Pennsylvania	83 (11.87)	Michael S Okun	28 (9.90)
Italy	422 (10.21)	University College London	71 (10.16)	Li Zhang	28 (9.90)
Germany	339 (8.20)	Karonlinska Institutet	65 (9.30)	Daniela Berg	27 (9.54)
Canada	299 (7.23)	University of Florida	62 (8.87)	Paolo Barone	26 (9.19)
Spain	248 (6.00)	Harvard Medicine School	57 (8.15)	Huifang Shang	25 (8.83)
France	232 (5.61)	Johns Hopkins University	56 (8.01)	Paul Krack	22 (7.77)
Australia	212 (5.13)	Baylor College Medicine	51 (7.30)	Bei Cao	22 (7.77)
The Netherlands	185 (4.48)	Columbia University	50 (7.15)	Ruwei Ou	20 (7.07)

**Figure 2.**
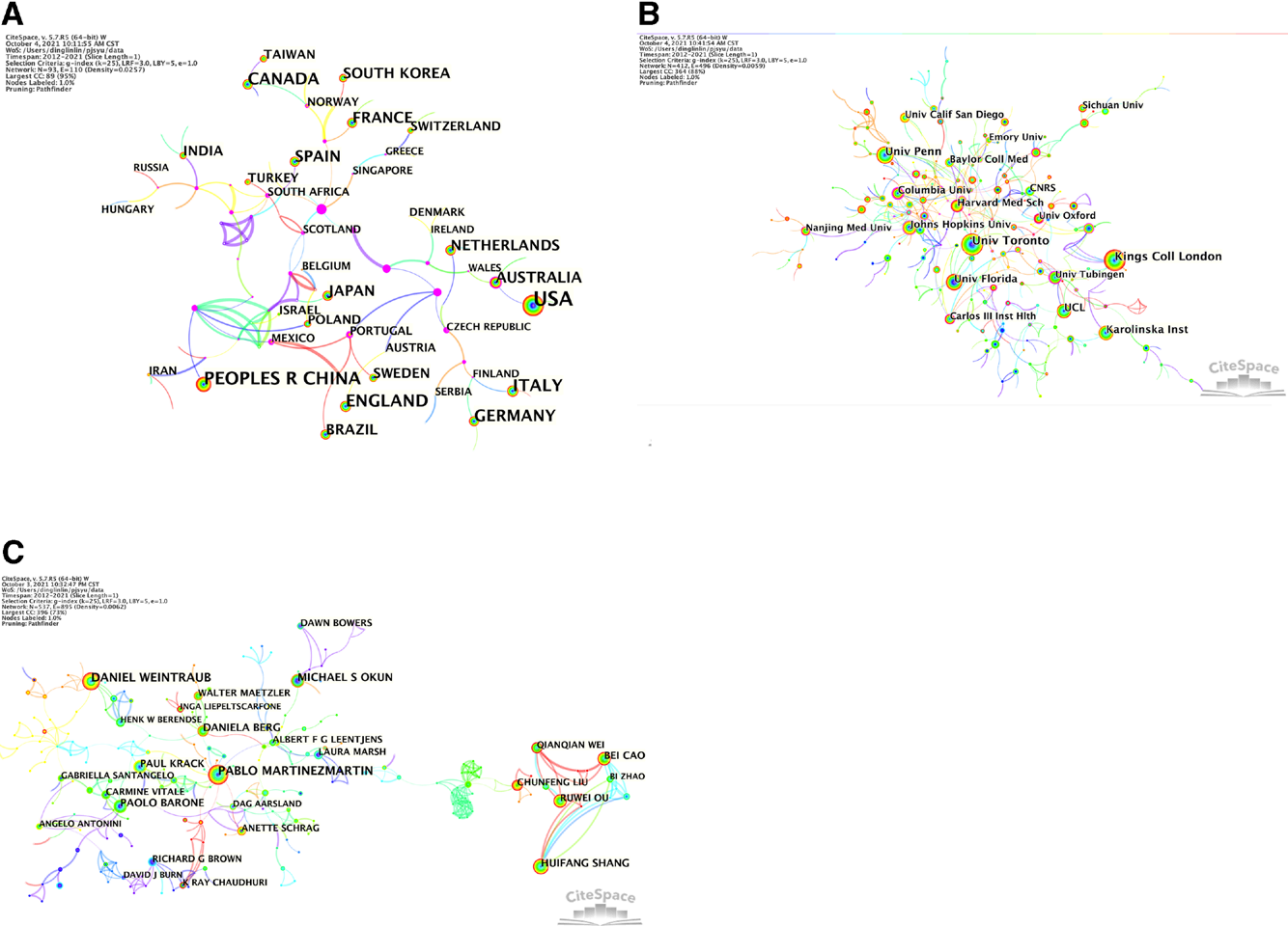
Visualization map of the scientific collaboration network analysis of depression in the Parkinson field from 2012 to 2021. Collaboration among countries (A), institutions (B), and authors (C).

Figure 2A indicates that the main research strength was at the universities. The United States has published the most studies and has conducted strong scientific research on depression in PD. Furthermore, the highest number of bursts in the study was in Iran, reaching 3.15. Iceland had the strongest centrality, reaching 0.7. The highest ranking from Sigma was for Thailand.

#### 3.1.3. Co-institution analysis

We ran CiteSpace, generating a network as usual: 2012–2021, slice length: 1 year; node selected the node type: institution, top N = 50, choice pathfinder, pruning sliced networks, and pruning the merged network. The other parameters were set to default settings. In addition, co-institution knowledge mapping was generated, in which N = 412 and E = 496 (density = 0.0059).

Figure 2B indicates that the main research strength was in the universities. The University of Toronto has published the most studies and has conducted strong scientific research on depression in PD. Furthermore, the highest number of bursts in the study was at the University of Roma La Sapienza, reaching 9.57. The University of Columbia had the highest centrality, reaching 0.3. The highest ranked by sigma was the University of Roma La Sapienza.

#### 3.1.4. Co-author analysis

By analyzing the author, cooperative relationships with others can be investigated. We ran CiteSpace, generating a network as usual: 2012–2021, slice length: 1 year; node select the node type: Author, Top N = 50, and choose Pathfinder, pruning sliced networks, and pruning the merged network; other parameter settings were likely to institutions. This study found knowledge mapping of the co-author with N = 537 and E = 859 (density = 0.0062).

Figure 2C shows that Daniel Weintraub ranked first in the number of publications, with 43 publications. The most obvious burst referred to David J Burn, reaching 5.31. The strongest centripetal force was observed for Pablo Martinezmartin, which displayed a centripetal force of 0.32. The highest Sigma (∑) was Laura Marsh, and the Sigma was 1.77.

### 3.2 Keyword cluster analysis

#### 3.2.1. Keywords analysis.

Keyword frequency analysis is an analytical approach to capture research hotspots, knowledge structures, and development trends in a subject. This analysis method is of great significance for understanding research trends. It can be seen from Table 2 that in terms of co-occurrence frequency, the top 10 keywords were “Parkinson’s disease”, “Depression”, “Quality of life”, “Dementia”, “non-motor symptom”, “Prevalence”, “Anxiety”, “Alzheimer’s disease”, “Symptom” and “Disorder”. According to the above keywords, the research hotspots of depression in PD can be summarized into 3 aspects: risk factors of depression in PD, assessment scale of depression in PD, and rehabilitation of depression in PD.

**Table 2:** The top 10 keywords in terms of frequency for depression in Parkinson disease field.

**Ranking**	**Keywords**	**Centrality**	**Frequency**
1	Parkinson disease	0	2224
2	Depression	0.03	1870
3	Quality of life	0.03	697
4	Dementia	0.02	564
5	Nonmotor symptom	0	465
6	Prevalence	0.02	462
7	Anxiety	0	457
8	Alzheimer disease	0	427
9	Symptom	0	395
10	Disorder	0.02	391

#### 3.2.2. Analysis of keywords clusters.

**Figure 3.**
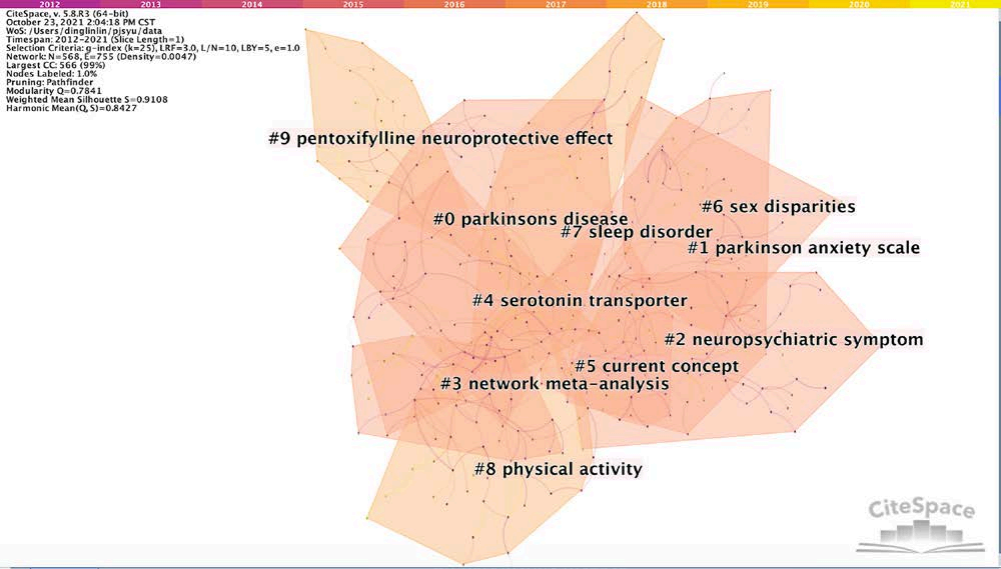
A landscape view of keyword cluster analysis generated by top 50 per slice from 2012 to 2021 (LRF = 3, LBY = 5, and e = 1.0. e = nodes, LBY = look back years, LRF = link retaining factor).

We ran CiteSpace, generating a network as usual: 2012–2021, slice length: 1 year; node select the node type: keyword; top N = 100 and choice path selection and pruning the merged network. Given the co-occurrence of keywords, the nodes were revised, and the log likelihood ratio algorithm was adopted for clustering calculations. The visualization map obtained N = 586, E = 755 (density = 0.0047), modularity Q score was 0.7841, and mean silhouette score was 0.9108, as shown in Figure 3.

There were a total of 10 clusters, as listed in Table 3. From Table 3, it can be seen that research hotspots in the field of depression in PD mainly focus on the Parkinson anxiety scale, neuropsychiatric symptoms, serotonin transporter, and pentoxifylline neuroprotective effects, among others. Most of the studies used network Meta-analysis to analyze the relationship quantitatively and qualitatively between depression and PD and to provide theoretical basis for the prevention and rehabilitation of PD. The results showed that sex disparities, sleep disorders, and physical activity could affect depression in PD.

**Table 3:** Subjects of cluster analysis (2012–2021).

**Clusters**	**Silhouette**	**Size**	**Log likelihood ratio**
#0 Parkinson disease	0.87	40	Umbrella review, managing chronic condition, opportunity cost
#1 Parkinson anxiety scale	0.886	37	Parkinson disease, life emotional distress, neuropsychological
#2 Neuropsychiatric symptom	0.93	37	Parkinson disease, health-related quality
#3 Network meta-analysis	0.956	33	Resting state, theta burst, randomized controlled study
#4 Serotonin transporter	0.837	33	Basal ganglia, serotonin neuron loss
#5 Current concept	0.903	32	Current knowledge, olfactory-related cortical atrophy
#6 Sex disparities	0.917	30	Cardiac event, national data-linkage study, all-cause mortality
#7 Sleep disorder	0.901	30	Depression anxiety, emotion processing, sleep quality
#8 Physical activity	0.934	29	Balance training, randomized controlled pilot trial, nonmotor function
#9 Pentoxifylline neuroprotective effect	0.949	29	TNF-alpha inhibitory properties, features alpha-synuclein

#### 3.2.3. Research hot spots and path analysis.

**Figure 4.**
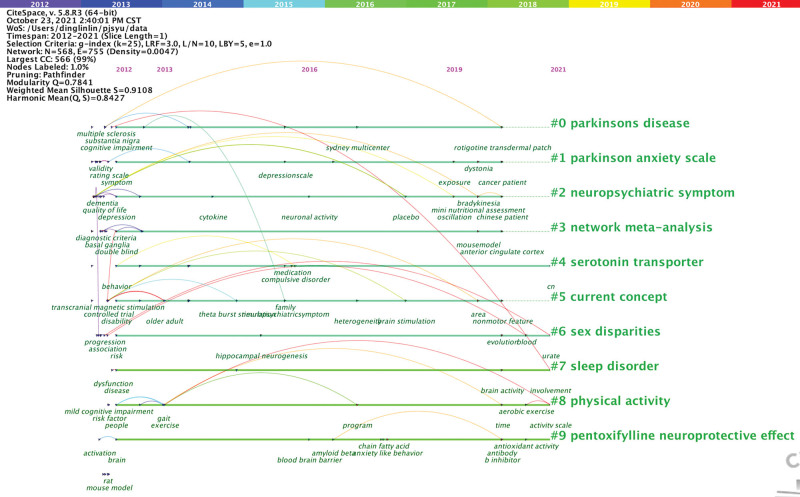
A time of the 14 largest clusters for depression in Parkinson disease (2012–2021).

Timeline visualization depicts clusters along a horizontal timeline. The main 10 clusters are shown in Figure 4. Each can indicate the evolution of research in the field of depression in PD from 2012 to 2021.

#### 3.2.4. Keywords citation bursts analysis.

**Figure 5.**
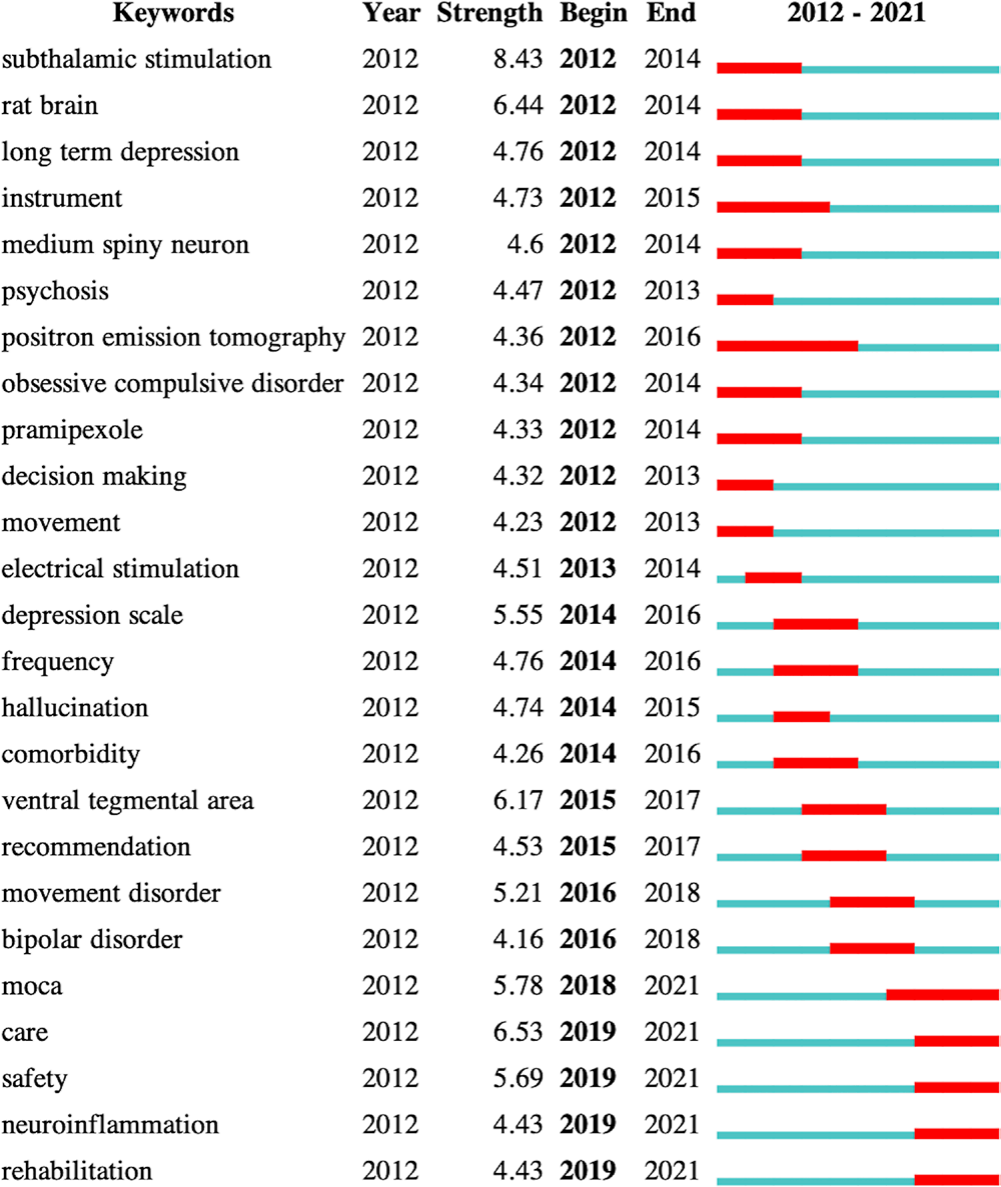
The evolution of the research topic in different periods (2012–2021).

Citation burst refers to keywords appearing suddenly in a short period or whose usage frequency increases sharply, revealing the evolution of the research topic in different periods, as listed in Figure 5.

## 4. Discussion

Combined with the high-frequency keywords and the detection results of sudden items, this study found that rehabilitation nursing and physical exercise are research hotspots and frontiers of Pd depression. PD is a neurodegenerative disease that places a heavy burden on the patients’ quality of life and independence. Neurorehabilitation training is an important method to improve the functional status of PD, especially the multidisciplinary nursing plan, which has shown positive effects on both motor and nonmotor symptoms.^[11,12]^ Multidisciplinary rehabilitation programs include motor, cognitive, and language training. Regular exercise should be encouraged, including gait and balance training,^[13]^ progressive resistance training,^[14]^ treadmill exercise,^[15]^ strength training,^[13]^ aerobic training,^[13]^ methods relating to music and dance,^[16]^ and tai chi.^[17]^ Different exercise methods may be beneficial for treating the different motor symptoms of PD. Cognitive training includes specific exercises for attention/working memory, psychomotor speed, executive function, visuospatial ability, computational skills, paper-and-pencil, and computerized tasks.^[18]^ In addition, physical, occupational, and speech therapies can also be useful.^[19]^ Therapeutic interventions can help maintain or improve motor symptoms, balance, gait, and function, and ameliorate hypovocalization and dysphagia. Multidisciplinary rehabilitation improved the functional status of PD patients and had a positive impact on mood, motor ability, autonomy of activities of daily living, perception of quality of life, cognitive ability, and speech skills.^[18]^ Referral and consultation with multidisciplinary treatment is an important part of high-quality care for PD.^[20]^

The construction of the Parkinson anxiety scale was the largest cluster in this study. Depression and anxiety are common psychiatric comorbidities of PD, which can lead to severe functional impairment, which can lead to poor quality of life.^[21]^ Mood disorders are often difficult to detect because their symptoms are similar to the cognitive and motor symptoms of PD. Therefore, the early detection and treatment of anxiety and depression are important for PD treatment. Anxiety disorders are more common in people with PD than in the general population or in people with other chronic diseases^[22]^; approximately one-third of patients experience a diagnosable anxiety disorder.^[23]^ The most common is general anxiety, typically characterized by persistent stress, irrational worry, and physical arousal, and phobias, especially social phobias and panic disorder.^[24]^ Dissanayaka et al^[25]^ reviewed the literature on PD anxiety scale and found that the 2 scales had good validity and reliability: The Geriatric Anxiety Inventory^[26]^ and The Parkinson Anxiety Scale.^[27]^ The types of depression that PD usually presents are^[4]^: major depression, major depression, bad mood, and subsyndromic depression. A meta-analysis showed that 3 depression scales were highly sensitive to Parkinson depression assessment^[28]^: the 15-item Geriatric Depression Scale,^[29]^ the Beck Depression Inventory,^[30]^ and the Montgomery-Asberg Depression Rating Scale.^[31]^

The burst item test results of this study showed that the Montreal Cognitive Assessment Scale^[32]^ is a hot spot and frontier in the current research on PD, which is one of the most common nonmotor symptoms of PD, and people with PD have a significantly increased risk of developing dementia compared to the general population. The prevalence of dementia in patients with PD is approximately 25% to 30%, and PD progresses to dementia if the patient survives for 10 years or more after the initial diagnosis.^[33]^ Depression in PD is characterized by severe cognitive problems that impair activities of daily living^[34]^; cognitive decline increases the risk of depression in PD^[35]^; therefore, cognitive diagnosis and intervention can prevent depression in PD. The cognitive score scale is useful for screening and monitoring in clinical practice, and the Dyskinesia Association Review Committee recommends the following 3 tools^[36]^: Montreal Cognitive Assessment, Montreal Cognitive Assessment Scale, Mattis Dementia Rating Scale Second Edition, Dementia Rating Scale Second Edition,^[37]^ and the PD-Cognitive Rating Scale.^[38]^ In a clinical setting, the most appropriate scale is the Montreal Cognitive Assessment Scale,^[39]^ because it only takes about 10 minutes to start, and it is sensitive to changes over time. A diagnostic score of <26 is recommended for PD mild cognitive impairment, and a score of <21 is recommended for depression in PD.^[40]^ As anxiety, depression, and cognition scales all come from abroad; there are some problems with cross-cultural adaptation and application. On the one hand, due to the differences between Chinese and Western cultures, it may be difficult to understand the same concept. On the other hand, most scales are self-reported and have subjective items, 1 dimension, and low specificity. Therefore, in terms of accuracy, assessments must be made by third parties (caregivers, e.g., family and medical professionals). Therefore, the current challenge for researchers is to improve the predictive validity, sensitivity, and specificity of the scale. Therefore, it is necessary to conduct large-sample, multicenter quantitative tests.

Combined with the high-frequency keywords and the detection results of sudden items, this study found that neuroinflammation are research hotspots and frontiers of PD depression. PD-associated depression is linked with many anatomical changes within the limbic system. The temporal cortex, particularly the amygdala and hippocampus, has been shown, in some studies, to be atrophic with negative correlation to depression severity, which could participate to mood/emotion learning deficits.^[41,42]^ Positron emission tomography [18]F-fluoro deoxy glucose and resting-state magnetic resonance imaging reveal that depression severity increases with amygdala metabolism increase.^[43,44]^ The amygdala plays a major role in integrating external stimuli and generating emotional responses; this could reflect excessive and uncontrolled emotion processing in depressed PD patients.^[44]^ The role of the orbitofrontal cortex in depression and anxiety in PD has also been particularly stressed in many magnetic resonance imaging studies showing notably atrophy of this region proportionally to the severity of depression.^[45,46]^ Finally, subcortical regions are also implicated in PD depression pathophysiology, in particular the limbic part of the thalamus, which has been found to be hypertrophic but also hypoactive during emotion perception.^[47]^ Furthermore, white matter abnormalities have been observed in the limbic thalamus, with reduced fractional anisotropy, proportional to depression severity.^[47]^

Depression in PD is a serious health problem for older adults that affects their physical and mental health and quality of life, as well as that of their families. This study introduced nearly a decade of research, from the rehabilitation nursing, physical exercise, anxiety and depression scale of evaluation, cognitive scale of evaluation and physiology of PD, and its association with depression. The latest progress in the study of this aspect is expounded and the related progress summed up the opinions of the experts, enabling more people to have a comprehensive understanding of PD and depression to reduce Parkinson depression-related harm in older adults. This study used scientific methods to find effective ways to prevent, treat, and reduce the risk of depression in PD. This study has some limitations. First, the selected studies are from the core journals of Web of Science, and the literature only contains English studies, so the current situation of the study cannot be extended to other language countries. Second, CiteSpace analysis tends toward quantitative analysis and qualitative research methods of interview methods should be adopted in subsequent research to make up for the defects of quantitative research.

## Author contributions

**Conceptualization:** Yan Liu, Linlin Ding

**Data curation:** Yan Liu

**Formal analysis:** Yunyan Xianyu

**Methodology:** Linlin Ding

**Software:** Yan Liu, Shuke Nie

**Writing—original draft:** Yan Liu

**Writing—review & editing:** Yunyan Xianyu, Shuke Nie

## Acknowledgments

The authors acknowledge Natural Science Foundation of Hubei Province (2021CFB451) to S.N. for partial support of this research.
